# The Impact of Simvastatin on Lipidomic Markers of Cardiovascular Risk in Human Liver Cells Is Secondary to the Modulation of Intracellular Cholesterol

**DOI:** 10.3390/metabo11060340

**Published:** 2021-05-25

**Authors:** Yvette L. Schooneveldt, Corey Giles, Michael F. Keating, Natalie A. Mellett, Aaron W. Jurrjens, Sudip Paul, Anna C. Calkin, Peter J. Meikle

**Affiliations:** 1 Metabolomics Laboratory, Baker Heart and Diabetes Institute, Melbourne, VIC 3004, Australia; yvette.schoonveldt@baker.edu.au (Y.L.S.); corey.giles@baker.edu.au (C.G.); natalie.mellet@baker.edu.au (N.A.M.); aaron.jurrjens@baker.edu.au (A.W.J.); sudip.paul@baker.edu.au (S.P.); 2 Central Clinical School, Faculty of Medicine, Nursing and Health Sciences, Monash University, Melbourne, VIC 3004, Australia; 3 Lipid Metabolism & Cardiometabolic Disease Laboratory, Baker Heart and Diabetes Institute, Melbourne, VIC 3004, Australia; michael.keating@baker.edu.au; 4 Baker Department of Cardiometabolic Health, University of Melbourne, Parkville, VIC 3010, Australia

**Keywords:** statins, cholesterol, low-density lipoprotein cholesterol, cardiovascular disease, targeted lipidomics, lipid metabolism

## Abstract

Statins are the first-line lipid-lowering therapy for reducing cardiovascular disease (CVD) risk. A plasma lipid ratio of two phospholipids, PI(36:2) and PC(18:0_20:4), was previously identified to explain 58% of the relative CVD risk reduction associated with pravastatin, independent of a change in low-density lipoprotein-cholesterol. This ratio may be a potential biomarker for the treatment effect of statins; however, the underlying mechanisms linking this ratio to CVD risk remain unclear. In this study, we investigated the effect of altered cholesterol conditions on the lipidome of cultured human liver cells (Hep3B). Hep3B cells were treated with simvastatin (5 μM), cyclodextrin (20 mg/mL) or cholesterol-loaded cyclodextrin (20 mg/mL) for 48 h and their lipidomes were examined. Induction of a low-cholesterol environment via simvastatin or cyclodextrin was associated with elevated levels of lipids containing arachidonic acid and decreases in phosphatidylinositol species and the PI(36:2)/PC(18:0_20:4) ratio. Conversely, increasing cholesterol levels via cholesterol-loaded cyclodextrin resulted in reciprocal regulation of these lipid parameters. Expression of genes involved in cholesterol and fatty acid synthesis supported the lipidomics data. These findings demonstrate that the PI(36:2)/PC(18:0_20:4) ratio responds to changes in intracellular cholesterol abundance per se, likely through a flux of the n-6 fatty acid pathway and altered phosphatidylinositol synthesis. These findings support this ratio as a potential marker for CVD risk reduction and may be useful in monitoring treatment response.

## 1. Introduction

3-hydroxy-3-methyl–glutaryl-coenzyme A reductase (HMGCR) inhibitors, commonly referred to as statins, are prescribed universally as the standard first-line lipid-lowering therapy [1]. Statins target the requisite and rate-limiting step in the cholesterol biosynthesis pathway, HMGCR, which facilitates the conversion of HMG-CoA to mevalonate. Considerable evidence, including angiographic trials, has shown the success with which statins reduce cholesterol levels and subsequently attenuate atherosclerotic lesions, decreasing the risk of primary and secondary cardiovascular events linked to atherosclerosis [2,3].

Due to the strong relationship between elevated low-density lipoprotein cholesterol (LDL-C) levels and coronary events, the beneficial effects of statins have been largely attributed to a reduction of circulating LDL-C [4]. By inhibiting cholesterol synthesis, statins activate homeostatic mechanisms that increase the transcription of key genes involved in cholesterol synthesis and uptake, such as HMGCR and the LDL receptor (LDLR). Indeed, large clinical trials such as 4S, CARE and LIPID, have demonstrated the ability of statins to increase circulating high-density lipoprotein cholesterol (HDL-C) and decrease LDL-C levels between 18–60% [5,6,7,8]. Results from the LIPID trial demonstrated the benefit of these effects, reporting that pravastatin treatment reduced the incidence of myocardial infarction by 29%, death from cardiovascular disease by 24%, stroke by 19% and coronary revascularisation by 20% over a 6 year period [7].

More recent preclinical investigations have suggested that statins mediate additional beneficial actions beyond their ability to lower LDL-C levels. Proposed mechanisms include a reduction in endothelial nitric oxide synthase, improvement and restoration of endothelial function, enhanced stability of atherosclerotic plaques and decreased oxidative stress and vascular inflammation [9,10,11]. These benefits beyond cholesterol lowering are diverse, affecting many biochemical and physiological pathways, and provide further evidence for the pleiotropic effects of statins.

In one of the largest lipidomic studies to date, Jayawardana and colleagues characterised changes in the plasma lipidome in response to pravastatin treatment to define the relationship between statin treatment, plasma lipids and risk reduction of future cardiovascular events in secondary prevention [12]. The authors reported that while the modulation of LDL-C levels accounted for 32% of the observed risk reduction for secondary coronary events, the ratio of two lipid species, phosphatidylinositol (PI(36:2)) and phosphatidylcholine (PC(18:0_20:4)) accounted for 58% of the risk reduction, thus accounting for the majority of the treatment effect of pravastatin in risk reduction. Importantly, the PI(36:2)/PC(18:0_20:4) lipid ratio mediated the relative risk reduction for cardiovascular events independent of changes in LDL-C. These novel findings support the hypothesis that statins modulate alternative lipid pathways independent of lowering LDL-C levels. Whilst the mechanisms involved remain unclear, data from the study suggests that modulation of the n-6 fatty acid pathway and PI synthesis may play a role. Indeed, understanding the mechanisms that drive these changes could not only yield important information regarding the cardioprotective actions of statins but also aid in identifying poor responders to treatment. Therefore, the aim of this study was to determine whether changes in the PI(36:2)/PC(18:0_20:4) lipid ratio are a downstream consequence of cholesterol lowering or a pleiotropic effect of statins.

## 2. Results

This study aimed to better understand the mechanisms by which statins modulate the PI(36:2)/PC(18:0_20:4) lipid ratio. To achieve this, we developed a cell-based model that reflected the statin-induced changes observed in the human plasma lipidome by Jayawardana et al. [12]. Hep3B cells were selected as the majority of lipids are predominantly synthesised within hepatocytes. Criteria to confirm the biological relevance of the model included a significant decrease in the lipid ratio PI(36:2)/PC(18:0_20:4), as well as free cholesterol and cholesterol esters (CE), indicating successful inhibition of cholesterol synthesis and validation of the key findings from Jayawardana et al. [12]. Changes in phosphatidylinositol (PI) species and species containing arachidonic acid (AA; 20:4) were also considered. Simvastatin was utilised as it induces lipid metabolic changes that are most reflective of those observed in the human trial [12]. Furthermore, as simvastatin is a second generation statin, it has a significantly improved efficacy in reducing LDL-C levels compared to the earlier statins, including pravastatin [13].

### 2.1. Effect of Simvastatin on the Lipidome

**Figure 1:**
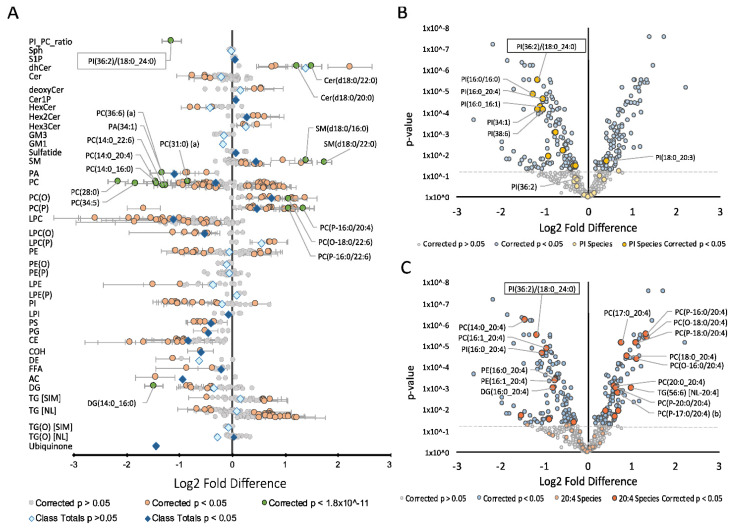
Effect of simvastatin treatment on the hepatic lipidome: Fold difference of 662 lipid species in Hep3B cells. Student’s *t*-tests were performed on 662 lipids following simvastatin treatment. Log2 fold difference denotes the change in (**A**) lipid abundance; (**B**) phosphatidylinositol species and (**C**) arachidonic acid (20:4) containing species with 5 μM simvastatin treatment. Data presented as log2 fold difference ±SD. *p*-values were corrected for multiple comparisons using Benjamini and Hochberg correction. Abbreviations: AC, acylcarnitine; CE, cholesteryl ester; Cer, ceramide; Cer-1-P, ceramide-1-phosphate; COH, free cholesterol; DE, dehydrocholesterol; deoxyCer, deoxyceramide; DG, diacylglycerol; dhCer, dihydroceramide; FFA, free fatty acid; GM1, GM1 ganglioside; GM3, GM3 ganglioside; HexCer, monohexosylceramide; Hex2Cer, dihexosylceramide; Hex3Cer, trihexosylceramide; LPC, lysophosphatidylcholine; LPC(O), lysoalkylphosphatidylcholine; LPC(P), lysoalkenylphosphatidylcholine; LPE, lysophosphatidylethanolamine; LPE(P), lysoalkenylphosphatidylethanolamine; LPI, lysophosphatidylinositol; NL, neutral loss; PC, phosphatidylcholine; PC(O), alkylphosphatidylcholine; PC (P), alkenylphosphatidylcholine; PE, phosphatidylethanolamine; PE(O), alkylphosphatidylethanolamine; PE(P), alkenylphosphatidylethanolamine; PG, phosphatidylglycerol; PI, phosphatidylinositol; PS, phosphatidylserine; PA, phosphatidic acid; SIM, single ion monitoring; SM, sphingomyelin; Sph, sphingosine; S1P, sphingosine-1-phosphate; TG, triacylglycerol; TG(O), alkyl-diacylglycerol.

Simvastatin treatment was associated with a significant decrease in cholesterol, total CE and the PI(36:2)/PC(18:0_20:4) ratio (Figure 1A). It was also associated with a smaller but significant decrease in phosphatidylcholine (PC) and a larger decrease in lysophosphatidylcholine (LPC) and lysoalkylphosphatidylcholine (LPC(O)) classes. Species within these classes containing either a 14:0, 16:0 or 16:1 fatty-acyl chain exhibited the most significant decrease. PC ether lipids, namely alkylphosphatidylcholine (PC(O)) and alkenylphosphatidylcholine (PC(P)) were increased in response to simvastatin treatment. In contrast, sphingolipids were less uniform in their response with some classes increasing whilst others decreased. Sphingomyelin (SM), sulfatide, ceramide-1-phosphate (Cer1P), dihexosylceramide (Hex2Cer) and sphingosine-1-phosphate (S1P) all increased and were the only sphingolipid classes to significantly change in response to simvastatin treatment. Other lipid classes including free fatty acids (FFA) and phosphatidic acid (PA) decreased, whilst diacylglycerol (DG) and triacylglycerol (TG) species demonstrated mixed responses. Specific analysis of PI species revealed that most PIs decreased after simvastatin treatment (Figure 1B), resulting in the observed minor decrease for the class overall. Lipid species containing AA together with 17:0, 18:0 or 20:0 fatty-acyl chain increased, while those combined with a 14:0, 16:0 or 16:1 fatty-acyl chain generally decreased (Figure 1C).

### 2.2. Effect of Alternative Cholesterol Modulating Treatment on the Lipidome

**Figure 2:**
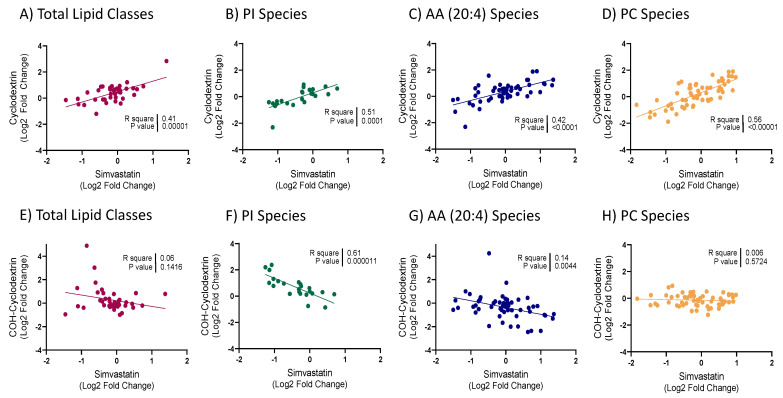
Correlation of changes in lipid species between treatments: Unadjusted linear associations between 5 μM simvastatin and 20 mg/mL cyclodextrin for (**A**) total lipid classes; (**B**) phosphatidylinositol (PI) species and (**C**) species containing arachidonic acid (AA; 20:4) and (**D**) phosphatidylcholine (PC) species; or 20 mg/mL COH-cyclodextrin for (**E**) total lipid classes; (**F**) phosphatidylinositol (PI) species; (**G**) species containing arachidonic acid (AA; 20:4) and (**H**) phosphatidylcholine (PC) species;log2 fold difference denotes the percentage between 5 μM simvastatin, 20 mg/mL cyclodextrin or 20 mg/mL COH-cyclodextrin treatments, relative to control. Data presented as log2 fold difference.

**Figure 3:**
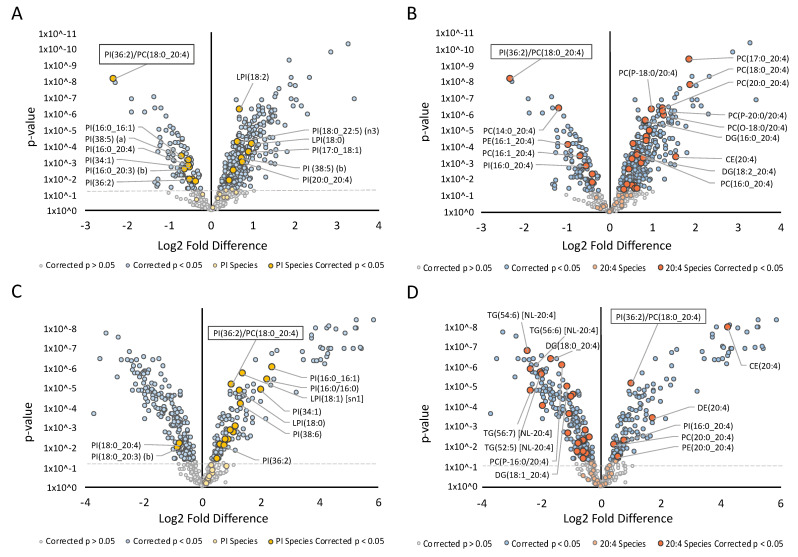
Effect of alternative cholesterol lowering treatments (cyclodextrin and cholesterol-loaded cyclodextrin) on the hepatic lipidome: Fold difference of 662 lipid species in Hep3B cells. Student’s *t*-tests were performed on 662 lipids following simvastatin treatment. Log2 fold difference denotes the change in (**A**) phosphatidylinositol species with 20 mg/mL cyclodextrin treatment; (**B**) arachidonic acid (20:4) containing species with 20 mg/mL cyclodextrin treatment; (**C**) phosphatidylinositol species with 20 mg/mL COH-cyclodextrin treatment and (**D**) arachidonic acid (20:4) containing species with 20 mg/mL COH-cyclodextrin treatment.

To investigate whether the effects of simvastatin on the lipid ratio were a downstream consequence of cholesterol lowering or the result of an off-target effect of simvastatin, cellular cholesterol levels were modulated using an alternative approach. Methyl-ß-cyclodextrin was utilised to deplete cells of membrane cholesterol, which resulted in a comparable, yet more exaggerated lipidomic profile to that seen with simvastatin (Figure S1). A strong concordance of the effects of simvastatin and cyclodextrin on levels of total lipid classes was observed (*p* = 0.00001; Figure 2A). Overall, the majority of lipid classes increased significantly in response to cyclodextrin treatment. Those that decreased included cholesterol, PC and PA, consistent with simvastatin treatment. Importantly, changes in individual PI and PC species were significantly correlated between simvastatin and cyclodextrin treatments (*p* = 0.0001; Figure 2B and *p* = 0.00001; Figure 2D). Specifically, PI(36:2), a constituent of the lipid ratio, as well as other PI species that contained a 16:0 or 16:1 fatty-acyl decreased (Figure 3A). Similarly, the majority of species containing AA (20:4), including the PC(18:0_20:4) constituent of the ratio, were modulated in a similar manner to simvastatin, increasing in response to cyclodextrin treatment (Figure 2C and Figure 3B).

To assess whether the addition of cholesterol would result in opposing effects to that observed when cells were depleted of cholesterol, cells were treated with the cholesterol donor, cholesterol-loaded cyclodextrin (COH-cyclodextrin). Interestingly, this did not appear to produce the same magnitude of effect on the lipidome as seen with simvastatin or cyclodextrin (Figure S2). Whilst the PI(36:2)/PC(18:0_20:4) lipid ratio, free cholesterol and CEs significantly increased, other classes that showed significant changes previously, in particular, sphingolipids and some glycerophospholipids, remained unchanged. CE, FFA and LPC classes exhibited the greatest increase in response to treatment, whilst TG classes, ubiquinone, PA and Hex3Cer were the only classes to significantly decrease. These lack of changes across almost all lipid classes resulted in an overall weak reciprocal correlation between COH-cyclodextrin and simvastatin treatments (Figure 2E). Interestingly, changes in PI and PC species as well as lipids containing AA showed opposing changes compared to those observed following simvastatin or cyclodextrin treatments (Figure 2F–H and Figure 3C,D).

### 2.3. Gene Expression

**Figure 4:**
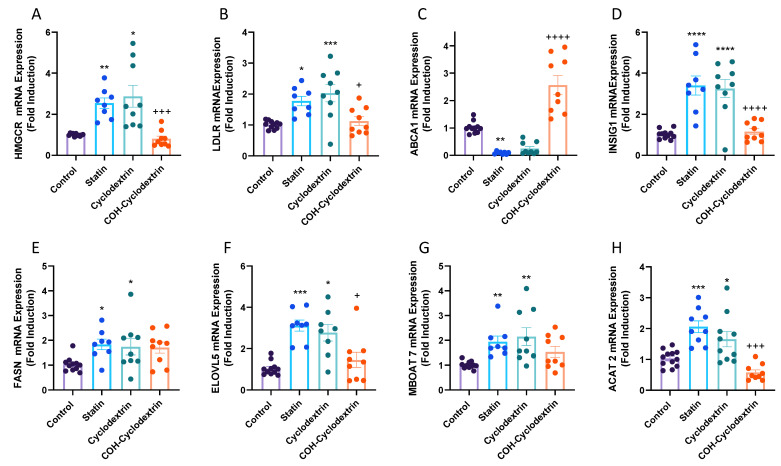
Expression of lipid regulating genes in response to cholesterol modulating treatments in Hep3B cells: (**A**) *HMGCR*; (**B**) *LDLR*; (**C**) *ABCA1*; (**D**) *INSIG1*; (**E**) *FASN*; (**F**) *ELOVL5*; (**G**) *MBOAT7* and (**H**) *ACAT2* mRNA expression relative to RPLP0 and expressed as fold change over control. Data are presented as mean ± SEM (*n* = 3/group with 2–4 technical replicates per experiment). Data were assessed for normality using a Shapiro–Wilk test and analysed using one-way ANOVA with Tukey post hoc testing for multiple comparisons. Non-parametric data was analysed using the Kruskal–Wallis test with Dunn’s multiple comparison test. * *p* < 0.05, ** *p* < 0.01, *** *p* < 0.001, **** *p* < 0.0001 vs. control; + *p* < 0.05, +++ *p* < 0.001, ++++ *p* < 0.0001 vs. statin. Abbreviations: *HMGCR*, 3-hydroxy-3-methylglutaryl-CoA reductase; *LDLR*, low density lipoprotein receptor; *ABCA1*, ATP-binding cassette transporter 1; *INSIG1*, insulin-induced gene 1 protein; *FASN*, fatty acid synthase 1; *ELOVL5*, elongation of very long-chain fatty acids protein; *MBOAT7,* membrane bound O-acyltransferase domain containing 7; *ACAT2,* acetyl-CoA acetyltransferase 2.

To gain further insight into the mechanisms underlying the observed changes across lipid classes, we assessed mRNA expression of key genes involved in relevant lipid signaling pathways. Expression of *HMGCR*, the rate-limiting enzyme in cholesterol synthesis, *LDLR*, a key receptor involved in LDL-C uptake, and *ABCA1*, a marker of cholesterol efflux, were assessed as readouts of the sterol regulatory element binding protein 2 (SREBP2) and liver X receptor (LXR) pathways, respectively. Expression of *INSIG1,* which resides in the endoplasmic reticulum and regulates cholesterol synthesis, and *ACAT2*, an enzyme that esterifies cholesterol, was also assessed. Both *HMGCR* and *LDLR* mRNA expression increased in response to simvastatin (*HMGCR*; *p* < 0.01 vs. control, *LDLR*; *p* < 0.05 vs. control) and cyclodextrin (*HMGCR*; *p* < 0.05 vs. control, *LDLR*; *p* < 0.001 vs. control), whilst expression decreased in response to COH-cyclodextrin compared to simvastatin (*HMGCR*; *p* < 0.001 vs. simvastatin, *LDLR*; *p* < 0.05 vs. simvastatin) (Figure 4A,B). Conversely, expression of *ABCA1* decreased when intracellular cholesterol levels were lowered via simvastatin (*p* < 0.01 vs. control) and significantly increased in response to COH-cyclodextrin (Figure 4C; *p* < 0.0001 vs. statin). Interestingly, mRNA expression of *INSIG1* was similar to that seen with *HMGCR* and *LDLR* (Figure 4D). Expression of fatty acid synthase (*FASN*), a SREBP-1c target gene, only slightly increased in response to elevated cholesterol (Figure 4E; *p* < 0.05 vs. control). *ELOVL5* is a protein involved in the elongation of very long-chain fatty acids. Specifically, it converts dihomo-γ-linolenic acid (20:3) into AA in the n-6 fatty acid pathway. Our data demonstrates an inverse relationship between *ELOVL5* mRNA expression and total cholesterol levels (Figure 4F), similar to that seen with *INSIG1,* as well as *HMGCR* and *LDLR*. *ELOVL5* mRNA levels increased with simvastatin (*p* < 0.001 vs. control) and cyclodextrin (*p* < 0.05 vs. control). Expression of *MBOAT7*, a gene involved in PI synthesis, showed an increase with simvastatin and cyclodextrin treatments compared to control (Figure 4G; *p* < 0.01 vs. control); however there was no significant difference in expression with COH-cyclodextrin treatment. Finally, expression of *ACAT2,* the enzyme involved in cholesterol esterification, was consistent with the other SREBP targets, as it increased in response to lowered COH environments and returned to baseline (control) when COH levels increased (Figure 4H). These data demonstrate that simvastatin and cyclodextrin modulate gene expression in a similar manner, whilst COH-loaded cyclodextrin drives an opposing effect. Furthermore, it is apparent that markers of the SREBP and LXR pathways were modulated to restore cholesterol levels. Changes in *FASN* and *ELOVL5* mRNA expression also suggest that reduced cholesterol levels cause a flux through the n-6 pathway, consistent with our lipidomic data (Figure 4E,F).

## 3. Discussion

Lipids are the major structural components of all organelle and cellular membranes. Whilst membranes are highly organised, they undergo constant remodeling in response to extra- and intra-cellular stimulation [14]. Simvastatin inhibits the synthesis of cholesterol, a major building block in cellular membranes, leading to changes in membrane composition. This was reflected in the lipidome as lipid species were altered in response to simvastatin treatment. Simvastatin significantly decreased the PI(36:2)/PC(18:0_20:4) lipid ratio in Hep3B cells, consistent with the findings of Jayawardana et al. [12]. Specifically, the majority of PI species decreased whilst the PC(18:0_20:4) constituent of the lipid ratio increased in both studies. Cholesterol and CE also decreased in our studies, validating our cell model. Expression of *ACAT2* further endorsed the lipidomics data, indicating that the reduced cholesterol and CE levels sparked an increase in *ACAT2* expression, likely in an attempt to recover the low CE levels. Many species containing AA increased after simvastatin treatment, suggesting a flux through the n-6 fatty acid pathway was occurring. PC and TG ether lipids ((PC(O), PC(P) and TG(O)), appeared to be the primary lipid classes containing AA species that increased. This is in line with their known function as storage depots for polyunsaturated fatty acids (PUFA), such as docosahexaenoic acid (DHA; 22:6) and AA [15,16,17]. DHA and AA give rise to anti-inflammatory molecules such as lipoxins, resolvins, and protectins, as well as inhibit the production of pro-inflammatory cytokines [18,19]. As many studies have demonstrated that statins elevate PUFA in very low-density lipoproteins (VLDL), LDL and HDL, modulation of these essential fatty acids may be an additional mechanism by which statins exert some of their pleiotropic cardioprotective effects [20]. Interestingly, of the AA containing lipids that decreased, the paired fatty acid (in the sn-1 position) was often myristic acid (14:0), palmitic acid (16:0) or palmitoleic acid (16:1). These are the major saturated and monounsaturated fatty acids (MUFA) produced by the de novo lipogenesis (DNL) pathway [21]. Experimental studies have demonstrated that palmitic acid induces endoplasmic reticulum stress, cellular apoptosis and activates pro-inflammatory pathways [22,23,24]. Thus, this decrease in DNL species reinforces the idea that statins achieve some of their beneficial effects through modulation of fatty acid metabolism.

Altering cholesterol conditions via different treatments (cyclodextrin and COH-cyclodextrin) provided insight into whether changes in the PI(36:2)/PC(18:0_20:4) ratio were an off-target effect of statins, or the consequence of cholesterol lowering. Cyclodextrin induces a low-cholesterol phenotype by specifically extracting cholesterol from the outer leaflet of the plasma membrane [25]. This mechanism of cholesterol reduction does not inhibit cholesterol biosynthetic pathways and therefore does not affect endogenously synthesised cholesterol nor any downstream metabolites. mRNA expression of genes involved in cholesterol synthesis and uptake (*HMGCR*, *LDLR*) confirmed a similar effect on endogenous cholesterol levels between simvastatin and cyclodextrin treatments. Hep3B cells treated with cyclodextrin demonstrated a comparable lipid profile to simvastatin. The levels of PI(36:2) and PC(18:0_20:4) decreased and increased respectively with cyclodextrin treatment, resulting in an overall decrease in the PI(36:2)/PC(18:0_20:4) ratio. A greater number of species containing AA increased after cyclodextrin, aligning with the ability of cyclodextrin to rapidly remove cholesterol from cellular membranes, faster than physiological cholesterol acceptors [26]. However, species with AA paired with palmitic or palmitoleic acid still decreased. As these effects were consistent between both cyclodextrin and simvastatin treatments, as well as the study by Jayawardana et al., it is evident that the PI(36:2)/PC(18:0_20:4) lipid ratio responds to changes in intracellular cholesterol abundance per se, rather than an off-target effect of statin treatment. Conversely, induction of a high cholesterol environment via COH-cyclodextrin resulted in a somewhat reciprocal lipid profile, including an increase in the PI(36:2)/PC(18:0_20:4) lipid ratio. COH-cyclodextrin inserts cholesterol back into cellular membranes, inducing a high-cholesterol phenotype. Elevated mRNA expression of *ABCA1*, an LXR target gene that promotes cholesterol efflux, and a significant increase in cholesterol and CE species, confirmed the success of the COH-cyclodextrin treatment. Most PI species increased after treatment whilst the majority of species containing AA decreased. Changes in AA species containing palmitic acid showed an opposing effect as they increased in response to COH-cyclodextrin treatment. This suggests an upregulation of de novo lipogenesis in response to the increased cellular cholesterol.

The underlying mechanisms driving this change in the PI(36:2)/PC(18:0_20:4) lipid ratio appear to be diverse and complex. The increase in species containing AA, including PC(18:0_20:4), across multiple lipid classes, suggests that modulation of the n-6 fatty acid pathway is occurring. Expression of *ELOVL5*, the enzyme that catalyses the first and rate-limiting step involved in the long-chain fatty acid elongation cycle of the n-6 pathway, supports this. Further analysis suggests that this flux is likely caused by the SREBP pathway. SREBPs are a family of membrane-bound transcription factors and are considered the primary regulators of cholesterol and unsaturated fatty acid synthesis [27]. Statins are known to upregulate transcription of all three SREBP isoforms (SREBP-1a, -1c and 2) within the first 24 h of treatment [28]. Using *ELOVL5^−/−^* mice, Moon and colleagues further demonstrated that endogenously synthesised PUFA are key regulators of SREBP-1c activation and fatty acid synthesis in the liver [29]. This is supported by a study demonstrating that mRNA expression of Δ5 desaturase, the rate-limiting enzyme responsible for the conversion of dihomo-γ-linoleic acid (20:3) to AA, was markedly upregulated by simvastatin via SREBP-1c [30]. This suggests that simvastatin may upregulate SREBP-1c within 8–24 h of treatment, resulting in a marked increase in PUFA and MUFA. Over time, however, as the end products of endogenously synthesised PUFAs’ (AA and DHA) feedback to suppresses SREBP-1c activity, SREBP-1c expression is subsequently downregulated [29]. This is consistent with our data showing a slight increase in mRNA expression of the SREBP-1c target gene *FASN*.

Whilst most PI species decreased in response to simvastatin and increased after COH-cyclodextrin, the underlying mechanisms remain unclear. Jayawardana et al. proposed that pravastatin reduced PI species via modulation of CDP-diacylglycerol synthesis, an important intermediate in phospholipid biosynthesis [12]. Work by Stamler et al. supports this, demonstrating that elevated PI species aid in the clearance of cholesterol and CE from the plasma [31]. Together, these findings indicate that lowering plasma cholesterol via simvastatin may inhibit PI synthesis in an effort to retain plasma cholesterol levels. Results from our study suggest that changes in fatty acid substrates available for PI synthesis may be an additional factor. This was made evident by the PI species that increased, as they all contained PUFA involved in the n-3 and n-6 fatty acid pathways. Furthermore, species containing linoleic acid (LA (18:2)), which is converted into AA, decreased. This suggests that the increase in n-6 fatty acids changes the proportion of fatty acid substrates available for PI synthesis. Combining this decrease in availability of LA and overall decrease in PI species may explain the stronger decrease in PI(36:2), resulting in the observed decrease in the lipid ratio. It is important to note that as cyclodextrin does not inhibit cholesterol biosynthesis, endogenous cholesterol is still synthesised. This results in the presence of low amounts of free cholesterol and may explain why some lipid classes that are typically sensitive to cholesterol levels, such as PI species, did not appear to be as strongly affected by the cyclodextrin treatment.

Whilst the majority of the findings in this study support the observations of Jayawardana et al., there are some differences to be noted. Jayawardana et al. observed a significant decrease in plasma TG species whilst our data demonstrated an increase in cellular TG species. As simvastatin drastically reduces cholesterol and CE levels, formation of VLDLs may be hindered. This would result in the observed accumulation of cellular TGs as their secretion is reduced. Differences between simvastatin and cyclodextrin treatments were also observed, however, these differences were consistent with their unique mechanisms of lowering cholesterol. For example, CE decreased after simvastatin treatment and increased with cyclodextrin. As discussed previously, cyclodextrin does not inhibit cholesterol synthesis. This means endogenous cholesterol is still available for esterification into CE, however, we would expect CE to gradually decrease due to hydrolytic activity of cholesterol esterase in an attempt to increase the cells’ depleted cholesterol levels. The magnitude of changes across various lipid classes in response to simvastatin and cyclodextrin treatments were also markedly different. This is consistent with the ability of cyclodextrin to rapidly reduce cholesterol levels by 80–90% [25]. Surprisingly, we did not observe the same exaggerated lipid profile with COH-cyclodextrin, suggesting that cholesterol removal has a greater impact on lipid metabolism than supplementation. It is also important to note that whilst the mechanism of action of each treatment is understood, various other mechanisms and feedback pathways may also be induced in response to each treatment. Subsequent modulation of these downstream pathways may have additional effects, contributing to the benefits of statins.

Our study has several limitations. As our cell model involved harvesting cells following a 48 h treatment, it is important to consider that many of the changes in lipids may not be in response to the simvastatin treatment, but rather in response to changes in feedback mechanisms to negate the effects of the cholesterol inhibition. Similarly, whilst Hep3B cells are human-derived liver cells, they are an immortalised cell line with known metabolic differences to animals and humans. Further validation studies in primary hepatocytes at multiple time points would be important in understanding the effect of statins on lipid abundance in cells with similar characteristics to an in vivo model. Additionally, this study focused on gene expression to validate flux through metabolic pathways, however, assessment of protein levels would offer further insight into the regulation of such pathways. Finally, the difference in potency between statins has been detailed extensively. In this study, we used simvastatin and whilst the results suggest this is a class effect of statins, alternate statins should also be tested to validate this.

## 4. Materials and Methods

### 4.1. Cell Culture

The immortalised human hepatoma cell line, Hep3B, was used for cell culture experiments (ATCC, Manassas, VA, USA). Hep3B cells were cultured in DMEM supplemented with 10% (*v/v*) Foetal Bovine Serum (Thermofisher, Waltham, MA, USA) and passaged every 2 to 3 days. Cells were passaged a maximum of 20 times before being discarded. Cells were maintained at 37 °C and 5% CO_2_. At 90% confluency, cells were dissociated using trypsin (Thermofisher, Waltham, MA, USA) and seeded at a density of 2.3 × 10^5^ cells per well in a 6-well plate for lipidomic analysis or 2.45 × 10^5^ per well in a 6-well plate for RNA isolation studies. Treatments were added 24 h after plating to ensure cells had adhered to the wells. Cells were harvested for analysis 48 h following the addition of treatments. Cell culture experiments for RNA isolation were repeated 3 times with 2–4 technical replicates per treatment. For lipidomics, the experiment was performed once with 6 technical replicates per treatment.

### 4.2. Treatment of Cells with Cholesterol Modulators

After plating for 24 h, Hep3B cells were washed in PBS without Ca^2+/^ and Mg^2+^ and treated with DMEM supplemented with 10% (*v/v*) lipoprotein-deficient foetal bovine serum (Thermofisher, Waltham, MA, USA) plus the relative treatment. Treatments were as follows: 5 µM simvastatin (Sigma Aldrich, St. Louis, MO, USA) dissolved in dimethyl sulfoxide (DMSO) (Sigma Aldrich, St. Louis, MO, USA) plus 10 µM mevalonate (Sigma Aldrich, St. Louis, MO, USA) dissolved in ethanol (Thermofisher, Waltham, MA, USA) (statin treatment), 20 mg/mL methyl-ß-cyclodextrin plus vehicle (0.1% DMSO and 0.02% ethanol) (cyclodextrin treatment) and 20 mg/mL cholesterol-loaded methyl-ß-cyclodextrin plus vehicle (0.1% DMSO and 0.02% ethanol) (COH-cyclodextrin treatment) or vehicle alone (DMSO and ethanol) (control). Methyl-ß-cyclodextrin and cholesterol-loaded methyl-ß-cyclodextrin (Sigma Aldrich, St. Louis, MO, USA) were prepared as follows: 5% methyl-ß-cyclodextrin (*w/v*) in H_2_O +/− 15 mg/mL cholesterol in 100% ethanol (methyl-ß-cyclodextrin: cholesterol at 10:1) was stirred for 30 min at 80 °C on a heat block before being dried down using a Savant SPD121P SpeedVac (Thermofisher, Waltham, MA, USA). Prior to experiments, treatments were freshly dissolved in molecular grade water.

### 4.3. Lipid Extraction

Hep3B cells were harvested in 200 µL of cold PBS on ice. Once collected, cells were disrupted by sonication with a Misonix S-4000 Sonicator (Misonix, Famingdale, NY, USA) for 10 s at amplitude 25. Protein concentrations were determined using a BCA assay, according to the manufacturer’s protocol (Pierce Protein Methods) (Thermofisher, Waltham, MA, USA) and as described by Brown et al. [32]. Aliquots of sample containing 50–60 µg of protein were then transferred into 1.5 mL microfuge tubes and dried down overnight in a Savant SPD121P SpeedVac (Thermofisher, Waltham, MA, USA), and resuspended in 10 µL PBS prior to extraction. Samples were then randomised to reduce bias prior to lipid extraction.

Lipids were isolated using a single phase chloroform:methanol (CHCl_3_/MeOH) (Merck, Kenilworth, NJ, USA) solvent extraction as described by Weir et al. [33]. Briefly, randomised cell lysates (10 µL) were combined with 200 µL CHCl_3_:MeOH (2:1) and 10 µL of the internal standard mix (Table S1) (Avanti Polar Lipids, Alabaster, AL, USA). Plasma quality control (PQC) samples (pooled plasma from 6 healthy individuals), reagent blanks (MiliQ-H_2_O) and technical quality control (TQC) samples (pooled PQC extracts) were distributed throughout the randomised samples (1:10) to measure variation in the extraction process and mass spectrometry analysis. Extracts were mixed for 10 min on a rotary mixer, sonicated in a water bath at room temperature for 30 min, left to stand on a bench for 20 min and then centrifuged at 15,000 rcf for 10 min at 20 °C. The supernatant was transferred to a 96-well plate and dried using a Savant SPD121P SpeedVac. Samples were reconstituted in a 1:1 mixture of water saturated butanol and methanol containing 10 mM ammonium formate. Samples were centrifuged at 1000 rcf for 5 min at 20 °C before the supernatant was transferred into glass vials with 0.2 mL micro-inserts for further analysis.

### 4.4. Liquid Chromatography Electrospray Ionisation Tandem Mass Spectrometry

Lipid analysis was performed by ultra-high-performance liquid chromatography, electrospray ionisation-tandem mass spectrometry (UHPLC-ESI-MS/MS) using an Agilent 1290 HPLC coupled to an Agilent 6490 triple quadrupole mass spectrometer. The settings were as follows: gas temperature 150 °C, gas flow 17 L/min, nozzle pressure 20 psi, sheath gas temperature 200 °C, sheath gas flow 10 L/min, capillary voltage 3500 V, nozzle voltage 1000 V. Liquid chromatography was performed on a Zorbax Eclipse Plus RRHD C18, 1.8 μM, 100 × 2.1 mm column (Agilent Technologies) using solvents A and B consisting of water:acetonitrile:isopropanol, 50:30:20 and 1:9:90 respectively, both containing 10 mM ammonium formate, with solvent A also containing 0.05 μM medronic acid. The column was heated to 45 °C and the auto-sampler regulated to 25 °C. Lipid extracts (1 µL) were injected and separated under gradient conditions with a flow rate of 400 µL/min. The gradient was as follows: 15% solvent B to 50% solvent B over 2.5 min, increase to 57% solvent B over 0.1 min, increase to 70% solvent B over 6.4 min, increase to 93% solvent B over 0.1 min, increase to 96% solvent B over 1.9 min, increase to 100% solvent B over 0.1 min, and hold at 100% solvent B for 0.9 min. Solvent B was then decreased to 15% over 0.2 min, held at 15% solvent B for 0.7 min, until next injection. Total time was 13.5 min with the first 1.2 min of each analytical run diverted to waste.

### 4.5. Quantification of Lipid Species

A total of 662 lipid species across 39 lipid classes were measured using dynamic multiple reaction monitoring (dMRM) where data was collected for a retention time window specific to each lipid species (Tables S3 and S4). Results from the chromatographic data were analysed using Mass Hunter Quant B9.0. Chromatographic peaks were integrated and assigned to a specific lipid species based on dMRM ion pairs and retention time. Lipid concentrations were calculated by relating each area under the chromatogram for each lipid species to the corresponding internal standard. Correction factors were applied to adjust for different response factors where these were known [34]. Lipid class totals were calculated as the sum of the individual species within each class.

### 4.6. RNA Isolation and Reverse Transcription-Polymerase Chain Reaction (RT-PCR) and Quantitative RT-PCR (qPCR)

Hep3B cells were harvested on ice in 500 µL of RNAzol (prepared in house) and transferred into 1.5 mL microfuge tubes. Samples were incubated at room temperature for 5 min before 100 µL chloroform (Merck, Kenilworth, NJ, USA) was added to phase separate the RNA. Samples were shaken rapidly, incubated for 3 min at room temperature, then centrifuged at 12,000 *rcf* for 15 min at 4 °C. The separated aqueous phase was transferred into a microfuge tube and 500 µL of room temperature isopropanol (Merck, Kenilworth, NJ, USA) was added. Samples were inverted 10 times and left to rest for 30 min at room temperature before being centrifuged at 14,000 *rcf* for 15 min at 4 °C. The supernatant was then discarded leaving an RNA pellet. The pellet was washed by adding 1 mL 75% (*v/v*) ethanol then centrifuged at 4 °C for 5 min at 12,000 *rcf*. The supernatant was removed before repeating these steps a further two times. Once the final wash had been completed, the pellet was centrifuged at 14,000 *rcf* for 1 min at 4 °C to remove any remaining ethanol. Pellets were then dried on a heat block at 55 °C for 10 min before resting on the bench for a further 5 min. The RNA pellet was resuspended in 12 µL of warmed molecular grade water and quantified using the NanoDrop (Thermofisher, Waltham, MA, USA) to assess RNA purity and quantity. Samples with 230/260 ratios above 1.8 and 260/280 ratios above 1.7 were used.

To generate cDNA, 2 µL (50 ng/µL) random hexamers (Scientifix, Melbourne, Australia) was added to 750–1000 ng RNA and samples were incubated at 70 °C for 5 min. After incubation, 5× first-strand buffer, 0.1 M DTT, 10 mM dNTP, 40 U/μL Ribosafe and 200 U/μL M-MLV reverse transcriptase (cDNA kit from Invitrogen, Carlsbad, CA, USA) were added, and samples were placed at 25 °C for 10 min, 37 °C for 50 min, and 70 °C for 10 min on the Applied Biosystems thermocycler. cDNA samples were then diluted with molecular grade water to 5 ng/μL for qPCR analysis in a 384-well plate. A master mix of SYBR Green (Invitrogen, Carlsbad, CA, USA), and the appropriate primer sets for target genes were prepared; 10 ng of cDNA was added to each well and qPCR was performed using the Applied Biosystems Quant Studio 7 real-time PCR machine. Conditions were as follows: Hold stage: 95 °C for 20 s PCR stage: 60 °C for 20 s; Melt Curve stage: 95 °C for 15 s, decrease to 60 °C for 1 min and increase back to 95 °C for 15 sec for 40 cycles. Quantification of a given gene was calculated using the ∆∆CT method. Data were normalised to the reference gene, Ribosomal Protein Lateral Stalk Subunit P0 (RPLP0), and expressed as fold change compared to the control group. Primer sequences were validated using the Basic Local Alignment Search Tool (BLAST) and are available in Supplementary Data (Table S2).

### 4.7. Data Presentation and Statistical Analysis

Samples for all mass spectrometry-based analysis were randomised prior to data acquisition. Quantification of lipid species was determined using R (3.4.0) analytical software. Lipid concentrations were normalised to total cellular protein content and log2 transformed prior to statistical analysis. Student’s *t*-tests were performed on 662 lipids. Mean differences and 95% confidence intervals were then converted to log2 fold difference for interpretation of results. Lipidomics data are presented as log2 fold difference ± SD. All *p*-values were corrected for multiple comparisons using the false discovery rate method of Benjamini and Hochberg [35]. qPCR data was assessed for normality using a Shapiro–Wilk test. Normally distributed data was analysed by one-way ANOVA with Tukey’s post hoc test. Non-parametric data was assessed using the Kruskal–Wallis test with Dunn’s test to determine significance between multiple groups. Individual data points were excluded due to technical inconsistencies in amplification curves. *p* < 0.05 was considered significant.

## 5. Conclusions

In summary, our findings have important implications for understanding the mechanisms underlying changes in the plasma PI(36:2)/PC(18:0_20:4) lipid ratio in response to statin treatment. We observed significant changes in multiple lipid species across 40 lipid classes with simvastatin treatment. Importantly, species containing AA increased, whilst free cholesterol, CE and the PI(36:2)/PC(38:4) ratio decreased. Replication of the low-cholesterol phenotype seen with simvastatin via the use of cyclodextrin resulted in a similar yet more exaggerated lipidomic profile. Conversely, the high cholesterol environment induced by cholesterol-loaded cyclodextrin exhibited a contrasting lipid profile. These data suggest that changes in intracellular cholesterol abundance and its downstream metabolites are the primary mediators of the lipid ratio, rather than a specific off-target effect of the statin itself. The mechanisms by which cholesterol mediates the constituents of the ratio PI(36:2) and PC(18:0_20:4) remain unclear, however, changes in *ELOVL5* and *FASN* gene expression suggest that it may involve modulation of the n-6 fatty acid pathway, via SREBP-1c, and inhibition of PI synthesis. Changes in the n-6 fatty acid pathway and subsequent increases in PUFAs may also explain some of the additional cardioprotective effects of statins. The PI(36:2)/PC(18:0_20:4) ratio may therefore serve as a putative surrogate marker for some of these pleiotropic effects on secondary outcomes, and could be useful in monitoring treatment response to statins. Despite the need for further investigations, it is clear that understanding these mechanisms may be important in uncovering the full treatment effect of statins.

## Data Availability

The data presented in this manuscript are available on request from the corresponding author. The raw data are not publicly available as particular software is required to open and view these data.

## Supplementary Materials

The following are available online at https://www.mdpi.com/article/10.3390/metabo11060340/s1, Table S1: Internal standards and mass spectrometry conditions used for lipid analysis in this study, Table S2: Primer sequence, Table S3: Effects of cholesterol modulating treatments on lipid classes in Hep3B cells, Table S4: Effects of cholesterol modulating treatments on lipid species in Hep3B cells, Figure S1: Effect of 20 mg/mL cyclodextrin on relative abundance of 662 lipid species in Hep3B cells, Figure S2: Effect of 20 mg/mL COH-cyclodextrin on relative abundance of 662 lipid species in Hep3B cells.
